# Reducing the Impact of Confounding Factors on Skin Cancer Classification via Image Segmentation: Technical Model Study

**DOI:** 10.2196/21695

**Published:** 2021-03-25

**Authors:** Roman C Maron, Achim Hekler, Eva Krieghoff-Henning, Max Schmitt, Justin G Schlager, Jochen S Utikal, Titus J Brinker

**Affiliations:** 1 Digital Biomarkers for Oncology Group National Center for Tumor Diseases (NCT) German Cancer Research Center (DKFZ) Heidelberg Germany; 2 Department of Dermatology and Allergy University Hospital LMU Munich Munich Germany; 3 Department of Dermatology Heidelberg University Mannheim Germany; 4 Skin Cancer Unit German Cancer Research Center (DKFZ) Heidelberg Germany

**Keywords:** dermatology, diagnosis, artificial intelligence, neural networks, image segmentation, confounding factors, artifacts, melanoma, nevus, deep learning

## Abstract

**Background:**

Studies have shown that artificial intelligence achieves similar or better performance than dermatologists in specific dermoscopic image classification tasks. However, artificial intelligence is susceptible to the influence of confounding factors within images (eg, skin markings), which can lead to false diagnoses of cancerous skin lesions. Image segmentation can remove lesion-adjacent confounding factors but greatly change the image representation.

**Objective:**

The aim of this study was to compare the performance of 2 image classification workflows where images were either segmented or left unprocessed before the subsequent training and evaluation of a binary skin lesion classifier.

**Methods:**

Separate binary skin lesion classifiers (nevus vs melanoma) were trained and evaluated on segmented and unsegmented dermoscopic images. For a more informative result, separate classifiers were trained on 2 distinct training data sets (human against machine [HAM] and International Skin Imaging Collaboration [ISIC]). Each training run was repeated 5 times. The mean performance of the 5 runs was evaluated on a multi-source test set (n=688) consisting of a holdout and an external component.

**Results:**

Our findings showed that when trained on HAM, the segmented classifiers showed a higher overall balanced accuracy (75.6% [SD 1.1%]) than the unsegmented classifiers (66.7% [SD 3.2%]), which was significant in 4 out of 5 runs (*P*<.001). The overall balanced accuracy was numerically higher for the unsegmented ISIC classifiers (78.3% [SD 1.8%]) than for the segmented ISIC classifiers (77.4% [SD 1.5%]), which was significantly different in 1 out of 5 runs (*P*=.004).

**Conclusions:**

Image segmentation does not result in overall performance decrease but it causes the beneficial removal of lesion-adjacent confounding factors. Thus, it is a viable option to address the negative impact that confounding factors have on deep learning models in dermatology. However, the segmentation step might introduce new pitfalls, which require further investigations.

## Introduction

**Figure 1:**
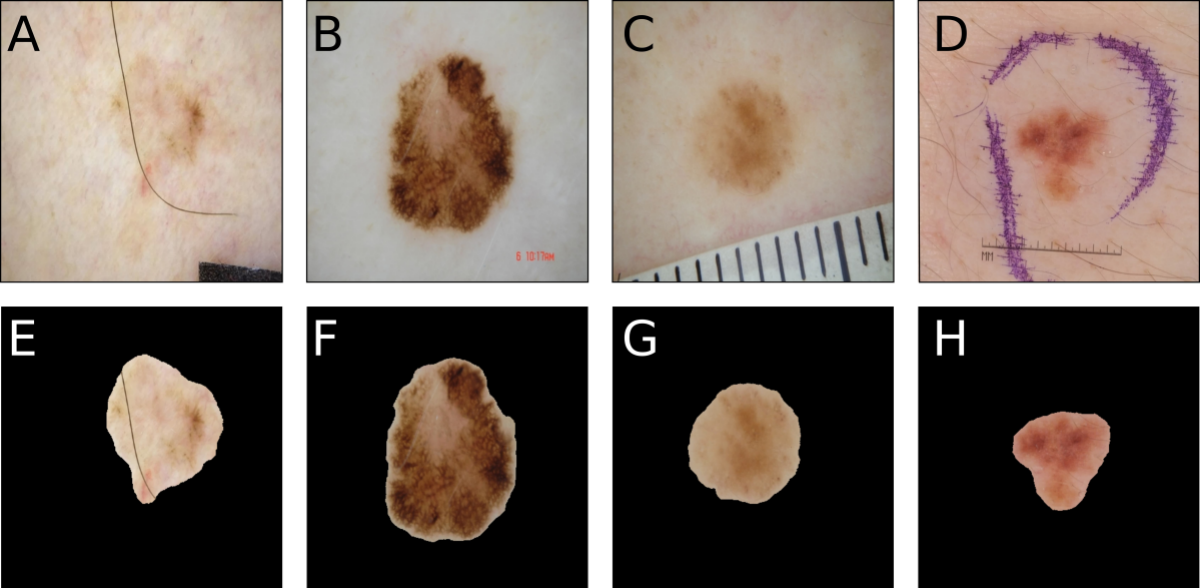
Typical artifacts encountered in dermoscopic image databases. Panels A-D show an exemplary range of artifacts often found in dermoscopic images, which are (left to right) color charts and hair, text, ruler markings, and marker ink. Panels E-H show how a corresponding segmented image could look like, with the surrounding artifacts removed but artifacts within the lesion (Panel E) still visible.

Deep learning models have achieved impressive results in dermoscopic image skin cancer classification, as exemplified by a range of studies on binary and multiclass classification tasks [1-5]. The creation of open-source dermoscopic image databases [6-8] has enabled much of the current research in this area by facilitating the training and evaluation of deep learning models. Supervised learning is commonly used, where the deep learning model is trained on labeled training data (eg, dermoscopic image plus its corresponding diagnosis), and it continually optimizes its internal parameters. This produces an inferred function that ideally classifies previously unseen data correctly based on a valid strategy (eg, in the case of skin lesions, based on relevant biological and structural features). However, it is not uncommon for deep learning models to learn spurious correlations within the training data. As a result, these models fail when evaluating data not exhibiting the respective correlations. In image analysis, such correlations are often introduced by visual artifacts, which act as confounding factors and have been observed to result in performance degradation [9,10]. A recent dermatology study showed that skin markings significantly interfered with the correct diagnosis of nevi by deep learning convolutional neural networks (CNNs) by increasing the melanoma probability scores and consequently, the false-positive rate [11]. Besides skin markings with stains/ink, a variety of artifacts are encountered in public and proprietary dermoscopic image databases, such as dark image corners, gel bubbles, color charts, ruler marks, or skin hairs (see Figure 1).

A variety of strategies have been proposed to tackle confounding factors such as digital hair removal, image cropping, or image segmentation [12]. In image segmentation, an image is partitioned into 2 or more regions so that each region can be analyzed on its own. Dermoscopic image segmentation usually partitions the image into foreground (lesion) and background (surrounding skin, see Figure 1). This preprocessing approach has the advantage that it not only simplifies the representation of the image but also removes the surrounding artifacts. Theoretically, the image fed to the deep learning model after segmentation consists mainly of the lesion, which presumably contains the most information but the least confounding factors.

In this study, we therefore determined if and how image segmentation affects skin lesion classification performance of deep learning–based algorithms. We compared the performance of 2 workflows: one where skin lesion classifiers were trained by a traditional end-to-end approach on unsegmented dermoscopic images and one where classifiers were trained by a two-step approach on images that have undergone prior segmentation.

## Methods

### Study Design

**Figure 2:**
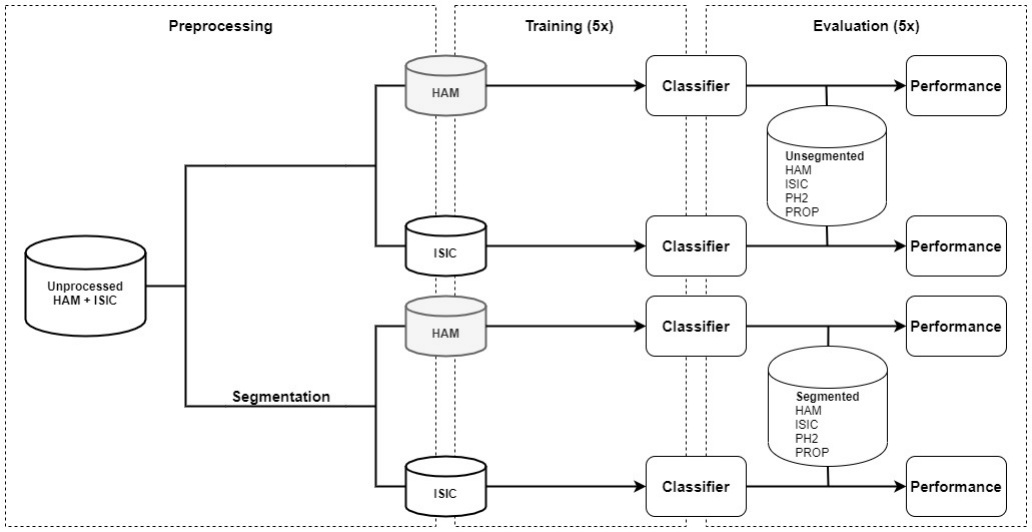
Flowchart of the study design. A training data set consisting of images from 2 different sources was either segmented or not segmented and split into 2 smaller partitions based on image origin (HAM or ISIC). An individual classifier was then trained on each of the 4 training sets and evaluated on a multi-source test set, which underwent a preprocessing step that equaled the training data preprocessing. Training and evaluation were repeated a total of 5 times for a more robust measure. HAM: human against machine data set; ISIC: international skin imaging collaboration data set; PH2: hospital Pedro Hispano data set; PROP: proprietary data set.

Binary classifiers (nevus vs melanoma) were trained on 2 different data sets and on unsegmented or segmented images, respectively, resulting in 4 separate types of classifiers. All classifiers were evaluated on a test set (n=688) consisting of 1 holdout component (n=200) and 3 external components (n=488). For each classifier type, 5 training and testing runs were performed in order to obtain robust performance estimates, which encompass the stochastic nature of the training process (see Figure 2). Ethics approval was waived by the ethics committee of the University of Heidelberg, as images were open source and anonymous.

### Data Sets

Dermoscopic images for developing the segmentation model were obtained from task 1 of the International Skin Imaging Collaboration (ISIC) 2018 challenge [7,13]. This data set is already split into a training, validation, and test set by the challenge organizers and contains dermoscopic lesion images together with a binary image mask, which partitions the image into a background (areas outside the primary lesion) and foreground (areas inside the primary lesion). This mask represents the “ground truth” with respect to the correct partitioning of the images. Dermoscopic images for developing the skin lesion classifiers were obtained from 2 sources: from part 3 of the ISIC 2017 challenge [6] and from the human against machine (HAM) data set [7]. Both data sets are mutually exclusive, with the HAM data set showing considerably fewer artifacts than the ISIC data set. Duplicated images within the HAM data set were removed prior to splitting the data set into the training, validation, and test set. The ISIC 2017 challenge data set had already been split by the challenge organizers.

Two additional external data sets were used for classifier evaluation. The first data set is publicly available and contains dermoscopic images acquired at the Dermatology Service of Hospital Pedro Hispano (PH2), Matosinhos, Portugal [8]. The second data set is proprietary (PROP) and contains dermoscopic images acquired from the Department of Dermatology and Allergy, University Hospital, LMU Munich, Munich and from the Department of Dermatology, Heidelberg University, Mannheim. Both data sets also contain some of the artifacts observed in ISIC and HAM, such as black image corners, rulers, or skin markings. As PH2 also contains binary image masks from dermatologists, this data set was also used for the evaluation of the segmentation model. Details on the training, validation, and test set composition are listed in Table S1 of Multimedia Appendix 1.

### Segmentation Model and Classifier Development

For image segmentation, a CNN in the form of a U-Net was employed [14]. The model’s raw output, which consists of a binary image mask, was further automatically processed by removing noise, closing holes, and replacing empty masks. Skin lesion classifiers were generated using a ResNet50 architecture, which was pretrained on ImageNet. For details on segmentation model and classifier development, refer to the supplementary methods (Multimedia Appendix 1).

### Analysis

The segmentation model’s performance was evaluated using a thresholded mean Jaccard index, a score between 0 and 1, which measures the similarity between the ground truth mask and the model’s output mask. The threshold was based on the ISIC 2018 challenge and set to 0.65, meaning that any lower scores were set to 0. The performance for each individual classifier was measured using balanced accuracy as the primary endpoint, with sensitivity, specificity, and area under the receiver operating characteristic curve (AUROC) as secondary endpoints. As we repeated each classifier training and evaluation step 5 times, metrics were first computed for each individual classifier and then averaged to obtain a mean performance measure. Performance comparisons were carried out between the preprocessing methods (ie, segmented vs unsegmented) and not between the underlying training data sets (ie, not HAM vs ISIC). Thus, we compared HAM segmented to HAM unsegmented and ISIC segmented to ISIC unsegmented but not HAM segmented to ISIC segmented. Statistical significance was evaluated for the primary endpoint by using a two-sided McNemar test and considered significant at *P*<.005 (Bonferroni correction by a factor of 10) to account for multiple testing when comparing the individual segmented HAM/ISIC classifiers to unsegmented HAM/ISIC classifiers for each of the 5 runs (one-on-one comparison). *P* values are listed only for significant runs.

## Results

### Segmentation Model Performance

The thresholded Jaccard index on the ISIC holdout test set for the segmentation model after mask postprocessing was 0.75 and increased to 0.81 on the external PH2 set.

### Classifier Performance

The overall balanced accuracy was numerically higher for the unsegmented ISIC classifiers (78.3% [SD 1.8%]) than for the segmented ISIC classifiers (77.4% [SD 1.5%]). This was significantly different in 1 out of 5 runs (*P*=.004). When trained on HAM, the segmented classifiers showed a higher overall balanced accuracy (75.6% [SD 1.1%]) than the unsegmented classifiers (66.7% [SD 3.2%]). This difference was significant for 4 out of the 5 classifiers (*P*<.001). A subanalysis of the performance on the holdout and external test set component shows that segmented classifiers had a numerically higher overall balanced accuracy on the external component than unsegmented classifiers, regardless of the data set source (see Table 1). The reverse trend was observed for the holdout component. AUROC followed the same trends as mean balanced accuracy.

**Table 1:** Overview of the balanced accuracy and area under the receiver operating characteristic curve for each type of classifier across the holdout, external, and overall test set.

Test set components, metric		Trained classifiers					
		HAM^a^ segmented (%)		HAM unsegmented (%)		ISIC^b^ segmented (%)	ISIC unsegmented (%)
**Holdout**							
	Balanced accuracy, mean (SD)		87.6 (1.4)		*89.4 (0.9)* ^c^	77.1 (1.5)	*80.0 (2.6)*
	AUROC^d^, mean (SD)		0.95 (0.006)		*0.964 (0.002)*	0.839 (0.008)	*0.89 (0.1)*
**External**							
	Balanced accuracy, mean (SD)		*69.9 (1.3)*		57.6 (4.1)	*78.2 (1.6)*	77.6 (1.7)
	AUROC, mean (SD)		*0.765 (0.011)*		0.647 (0.025)	*0.874 (0.005)*	0.851 (0.018)
**Overall**							
	Balanced accuracy, mean (SD)		*75.6 (1.1)*		66.7 (3.2)	77.4 (1.5)	*78.3 (1.8)*
	AUROC, mean (SD)		*0.841 (0.008)*		0.763 (0.02)	0.856 (0.005)	*0.862 (0.014)*

^a^HAM: human against machine data set.

^b^ISIC: International Skin Imaging Collaboration data set.

^c^The italicized data indicate the higher metric when comparing between classifiers trained on a segmented/unsegmented version of the same data set.

^d^AUROC: area under the receiver operating characteristic curve.

ISIC classifiers (regardless of preprocessing) show a comparable balanced accuracy across the holdout and external test set components, resulting in a similar balanced accuracy for the overall test set. In contrast, the segmented HAM classifiers show a substantially higher overall balanced accuracy to the unsegmented HAM classifiers. This better overall balanced accuracy stems from a visible performance difference on the external test set component, which is largely driven by a drop in the balanced accuracy for PH2. Here, the balanced accuracy of unsegmented HAM classifiers was 63.2% (SD 7.1%) compared to 84.4% (SD 2.9%) for the segmented HAM classifiers (see Table 2). Equivalent tables showing the results for the metric sensitivity and specificity are found in Table S2 and Table S3 of Multimedia Appendix 1.

**Table 2:** Overview of the balanced accuracy and area under the receiver operating characteristic curve for each type of classifier across the external test set’s 3 individual components.

External test set components, metric			Trained classifiers						
			HAM^a^ segmented (%)		HAM unsegmented (%)		ISIC^b^ segmented (%)		ISIC unsegmented (%)
**HAM/ISIC^c^**									
	Balanced accuracy, mean (SD)	*61.0 (1.3)* ^d^		58.9 (3.1)		74.1 (3.6)		*76.5 (1.8)*	
	AUROC^e^, mean (SD)	0.628 (0.005)		*0.636 (0.023)*		0.827 (0.019)		*0.851 (0.022)*	
**PH2^f^**									
	Balanced accuracy, mean (SD)	*84.4 (2.9)*		63.2 (7.1)		*86.4 (1.3)*		83.7 (0.8)	
	AUROC, mean (SD)	*0.928 (0.022)*		0.894 (0.021)		*0.947 (0.007)*		0.912 (0.018)	
**PROP^g^**									
	Balanced accuracy, mean (SD)	71.1 (1.8)		*75.7 (4.2)*		68.7 (1.6)		*74.6 (2.8)*	
	AUROC, mean (SD)	0.825 (0.033)		*0.857 (0.034)*		*0.88 (0.025)*		0.814 (0.015)	

^a^HAM: human against machine data set.

^b^ISIC: International Skin Imaging Collaboration data set.

^c^If classifiers were trained on HAM images, the first external test set component consists of ISIC and vice versa.

^d^The italicized data indicate the higher metric when comparing between classifiers trained on a segmented/unsegmented version of the same data set.

^e^AUROC: area under the receiver operating characteristic curve.

^f^PH2: hospital Pedro Hispano data set.

^g^PROP: proprietary data set.

### Additional Analyses

**Figure 3:**
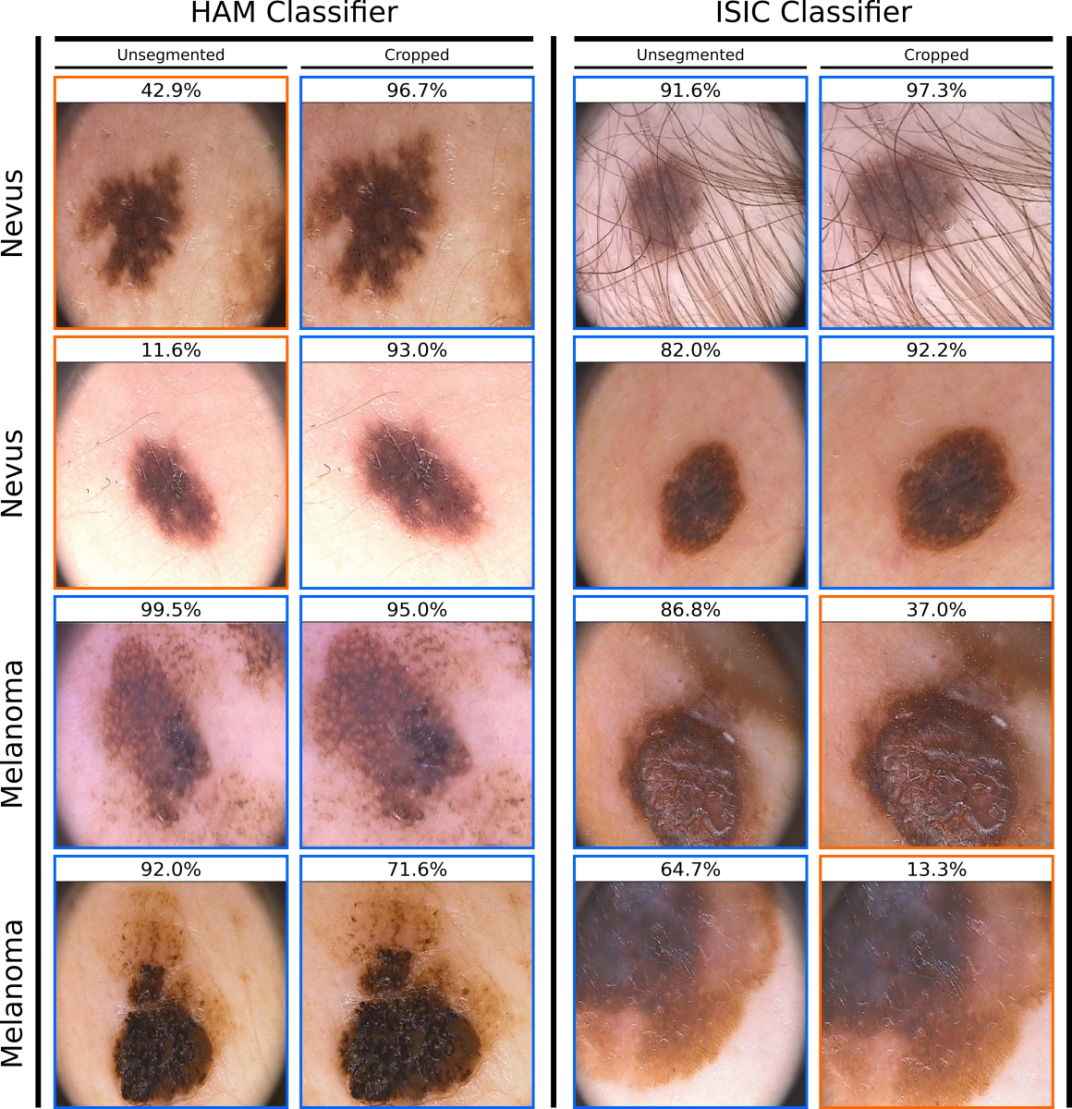
Exemplary predictions of a classifier trained on unsegmented HAM (left) and ISIC (right) images and evaluated on unsegmented and cropped PH2 images. The target class (ground truth) for each lesion is displayed to the left, with the classifier’s output probability for the target class on top. An output probability larger than 50% corresponds to a correct classification, which is also indicated by a blue frame, whereas an orange frame denotes an incorrect classification. HAM: human against machine data set; ISIC: international skin imaging collaboration data set; PH2: hospital Pedro Hispano data set.

Some additional analyses were carried out based on the obtained results. As the unsegmented HAM classifiers showed poor performance on PH2 with high sensitivity (95.5% [SD 1.9%]) but low specificity (30.9% [SD 13.6%], Table S3 of Multimedia Appendix 1), their performance was again evaluated on cropped unsegmented PH2 images. As the PH2 data set consists of images with predominantly black corners (see Figure 3), we speculated that these could be artifacts, which caused the drop in performance. We therefore manually cropped all unsegmented PH2 images just enough so that any black corner was removed. On cropped PH2 images, specificity increased to 65.8% (SD 8.3%) at almost unchanged sensitivity of 93.5% (SD 3%), resulting in an overall mean balanced accuracy of 79.6% (SD 3.8%). As the unsegmented ISIC classifiers showed a comparable performance to the segmented ISIC classifiers, there was no reason to assume that these classifiers are also negatively influenced by black image corners. However, when its performance was evaluated on cropped PH2, sensitivity decreased from 82% (SD 2.9%) (unsegmented) to 67.5% (SD 5.7%) (cropped) with specificity increasing from 85.4% (SD 2.8%) to 89.4% (SD 1.7%), resulting in a change of mean balanced accuracy from 83.7% (SD 0.8%) to 78.4% (SD 3.6%).

As ground truth segmentation masks were available for the PH2 data set, PH2 images were experimentally segmented using these masks instead of the masks produced by the segmentation model and subsequently used for evaluation. These masks were produced by an expert dermatologist; therefore, a similar performance was expected. However, segmented HAM and ISIC classifiers showed a lower balanced accuracy for PH2 images when processed by the ground truth masks (82% [SD 2.2%] and 76.1% [SD 2.9%], respectively) as opposed to the segmentation model mask (84.4% [SD 2.9%] and 86.4% [SD 1.3%], respectively). This change resulted from a drop in specificity from 80.2% (SD 3.8%) and 82.4% (SD 5.0%) to 76.5% (SD 5.6%) and 63.2% (SD 9.7%) at almost constant sensitivity (88.5% [SD 5.1%] and 90.5% [SD 3.7%] vs 87.5% [SD 5.0%] and 89.0% [SD 5.1%], respectively).

## Discussion

### Overview of the Study

In this study, we established and compared the performance of 2 classification workflows. The first workflow did not include a preprocessing step, and training and test set images were unmodified. The second included preprocessing where images were segmented prior to classifier training and evaluation. For training, we used 2 distinct training data sets (HAM and ISIC) and established the performance on a multi-source test set. Our findings show that while performance is highly dependent on the source of the training and test set, segmentation does not lead to an overall decrease in the performance of a ResNet50 architecture and may even lead to an improved classifier, which is presumably at a decreased risk to suffer from common lesion-adjacent confounding factors.

### Principal Results

The overall comparable performance of classifiers trained on segmented and unsegmented images shows that classifiers are able to distinguish melanoma from nevus images based largely on the lesion itself without requiring the surrounding skin area for additional information. This is not unexpected as visual inspection by dermatologists also mainly focuses on global and local features within the lesion, although features such as increased vascularization of the surrounding skin and lesions on sun-damaged or aged skin are associated with a higher risk of skin cancer and can thus be used as cues. While segmentation requires an extra step compared to end-to-end classification, it may be worthwhile as proper segmentation removes potential preexisting confounding factors surrounding the lesion (albeit not within the lesion, eg, hairs, overlapping rulers). Given the prevalence and large variety of artifacts in public dermoscopic databases such as ISIC, such measures are warranted to counteract the possibility of the classifier incorporating confounding factors in its decision process. For example, gentian violet skin markers were previously shown to be associated with a higher melanoma probability by a CNN approved for use as a medical device in the European market [11]. As artifact perception by a CNN-based classifier is dependent on the constitution of the underlying training data, this finding is not necessarily applicable to other CNN-based classifiers but highlights the negative impact of artifacts that may manifest themselves in a variety of ways. In this study, we hypothesize that classifiers trained on unsegmented HAM and ISIC images correlate black image corners with the occurrence of melanoma, albeit to varying degrees. Both unsegmented classifiers were evaluated on unsegmented and cropped PH2 images, where cropping completely removed the black corners (see Figure 3). In both cases, specificity increased when using cropped PH2 images. Sensitivity remained almost unchanged for the HAM classifiers and decreased for the ISIC classifiers, suggesting that classifiers trained on either training set associate black image corners with melanoma, but weigh its importance differently. Alternatively, it cannot be ruled out that the observed performance change stems from the cropping process, which introduces resolution changes, image distortions, and the removal of potentially relevant biological information if parts of the lesion are cropped out. However, given the one-sided performance increase (ie, for specificity) for classifiers from both data sets and the large prevalence of black image corners in the HAM and ISIC training data, a correlation is not unlikely.

As the segmentation step lies upstream of the classifier training and evaluation steps, the latter two are highly dependent on the output quality of the former. While the model employed in this study achieved a threshold Jaccard index lower than the score obtained by the ISIC 2018 challenge winners (0.75 vs 0.80), a general visual inspection of the segmentation masks suggested sufficient quality (ie, lesions visible with large portions of the background adequately removed). Further evaluation of its performance on an external data set (PH2) indicated that the segmentation model generalizes well and can be employed for segmenting images from external data sets. Assuming that classifier performance is partially indicative of segmentation performance, the segmentation model generalized adequately for HAM and PH2 images (known of course for the latter already due to the ground truth masks, but confirmed here again). In contrast, classifiers trained on segmented images performed worse on PROP with low mean balanced accuracies. Given that classifiers trained on unsegmented images did not suffer from this issue, insufficient segmentation masks are a possible candidate for the problem. This is, however, difficult to verify due to the nonexisting ground truth masks. This illustrates that the performance of segmented classifiers is ultimately tied to the performance of the segmentation model.

**Figure 4:**
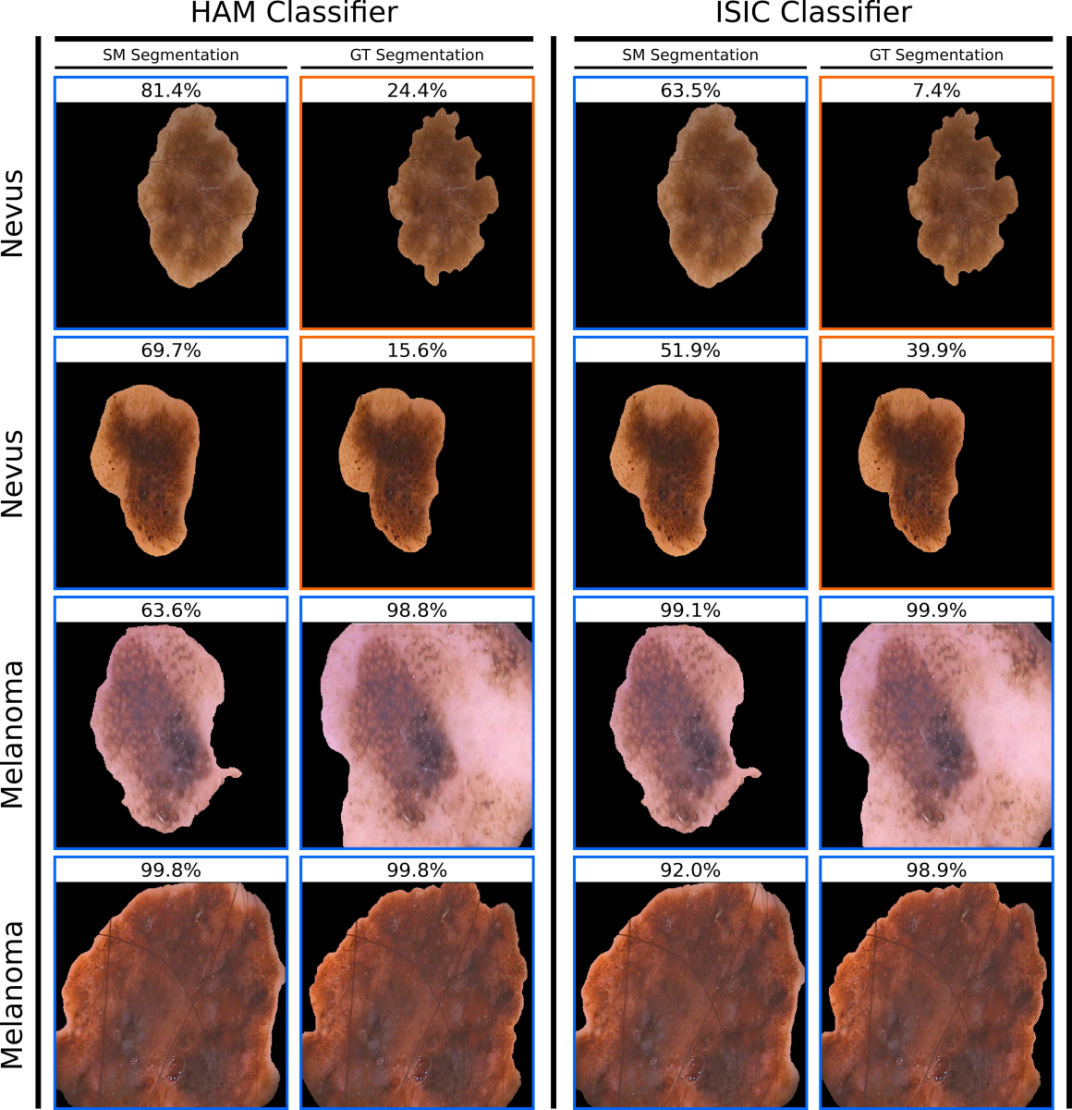
Exemplary predictions of a classifier trained on unsegmented HAM (left) and ISIC (right) images and evaluated on PH2 images with different segmentation masks. PH2 images in the SM column were segmented using the segmentation model. PH2 images in the GT column were segmented using dermatologist-generated ground truth segmentation masks. The target class (ground truth) for each lesion is displayed to the left, with the classifier’s output probability for the target class on top. An output probability larger than 50% corresponds to a correct classification, which is also indicated by a blue frame, whereas an orange frame denotes an incorrect classification. GT: ground truth; HAM: human against machine data set; ISIC: international skin imaging collaboration data set; PH2: hospital Pedro Hispano data set; SM: segmentation model.

In practice, identifying and fixing obviously faulty segmentation masks manually at test time should be feasible but may not be sufficient. As seen for the analysis of the PH2 set, where model segmentation masks were compared to ground truth segmentation masks, classifier performance may be strongly influenced by the precise way that the segmentation is done, with small differences causing large negative effects (see Figure 4). Training sets of automatically segmented images could contain their own kind of artifacts introduced by the automated segmentation process. We speculate that masks produced by the segmentation model have distinctive visual characteristics based on its training set and postprocessing methods. For instance, a certain amount of the adjacent skin may be included or the segmentation creates unique borders (eg, smoothness of edges). Any classifier trained on images with such segmentation masks might pick up on such subtleties and become susceptible to segmentation masks of a different variety.

### Future Work and Limitations

While the study aimed at only comparing the performance of 2 classification workflows where classifiers were trained on segmented/unsegmented images, there is notable performance variation dependent on the training and test sets. While the HAM training set contained more unique melanoma lesions (514 vs 374, Table S1 of Multimedia Appendix 1), the ISIC training data set contained more images of biopsy-verified lesions and thus, probably more borderline cases. These distinct features may be advantageous or detrimental for classifier performance on any given test set. Future work should address the issue of faulty segmentation masks and closely investigate the potential artifacts arising from an upstream segmentation step. As classification was done in a binary instead of a multi-class setting due to limited data availability, these findings might not generalize to a multi-class setting. Furthermore, performance was only shown here for 1 architecture; thus, generalizability to similar architectures, while expected, is not guaranteed.

### Conclusion

Skin lesion classifiers trained and evaluated on segmented images have an overall comparable performance to classifiers trained and evaluated on unsegmented images that show the exact same lesion. In addition, segmentation comes with the added benefit of removing lesion-adjacent artifacts, which may act as confounding factors. However, this benefit comes at a cost, as classifier performance is tied to the segmentation quality. Further, image segmentation may introduce new pitfalls. Hence, further investigation is required to elucidate the effects of segmentation observed in this study.

## Appendix

Multimedia Appendix 1 Supplementary content.

## Abbreviations

AUROC: area under the receiver operating characteristic curve
CNN: convolutional neural network
HAM: human against machine
ISIC: International Skin Imaging Collaboration
PH2: hospital Pedro Hispano
PROP: proprietary data set
