# Supplementary material for: Reducing the Impact of Confounding Factors on Skin Cancer Classification via Image Segmentation: Technical Model Study
Source: J Med Internet Res. 2021 Mar 25;23(3):e21695. doi: 10.2196/21695 (PMC8074854; doi:10.2196/21695)
Supplement: Multimedia Appendix 1 [file jmir_v23i3e21695_app1.docx]

Multimedia Appendix 1

**Table S1. Train, validation and test set class distribution for each dataset source.** Counts show the number of unique images. For the segmentation model development, train, validation and test images were not divided into melanoma and nevus classes. For the classifier model development, melanoma and nevus images were over- and undersampled respectively to create balanced classes for classifier training, therefore not all images listed here were used for training.

| **SEGMENTATION MODEL** | | | | | | |
| --- | --- | --- | --- | --- | --- | --- |
| **Dataset** | **Train** | | **Validation** | | **Test** | |
| **ISIC** | 2594 | | 100 | | 1000 | |
| **PH2** | 0 | | 0 | | 200 | |
| **CLASSIFIERS** | | | | | | |
| **Dataset** | **Train** | | **Validation** | | **Test** | |
|  | **Melanoma** | **Nevus** | **Melanoma** | **Nevus** | **Melanoma** | **Nevus** |
| **HAM** | 514 | 5303 | 0 | 0 | 100 | 100 |
| **ISIC** | 374 | 1254 | 47 | 348 | 100 | 100 |
| **PH2** | 0 | 0 | 0 | 0 | 40 | 160 |
| **PROP** | 0 | 0 | 0 | 0 | 37 | 51 |

**Supplementary Methods. Segmentation Model and Classifier Development.**

Training of the segmentation model and the classifiers shared many characteristics. For both, images were resized to 448x448 pixels and normalized using ImageNet’s mean and standard deviation [[1]](https://paperpile.com/c/aE78Ly/kOEX). Some online data augmentation was applied during training (random vertical & horizontal flips, brightness, hue, saturation) and learning rate and momentum were scheduled following Leslie Smith’s 1cycle policy [[2]](https://paperpile.com/c/aE78Ly/tbpC). Each training run consisted of two steps. In the first step (s1) only the network's upsampling layers (segmentation) or fully connected layers (classification) were trained with a single learning rate, while in the second step (s2), the complete network was trained with a discriminative learning rate. The training procedure was established by trial-and-error and optimized using a validation set. For the U-Net and the ResNet50 architecture the fastai library’s implementations were used [[3]](https://paperpile.com/c/aE78Ly/4ALs).

The segmentation’s model contracting path consisted of a ResNet34 with batch normalization layers. The model was trained for a total of 40 epochs (s1 = 5 epochs, s2 = 35 epochs) using a batch size of 8. Learning rate for the first step was set to 1e-3 and for the second step as a slice from 1e-6 to 1e-4. Segmentation masks generated by the model were further automatically processed by a variety of steps. First, holes within the foreground (lesion) were filled using SciPy’s binary_fill_holes function [[4]](https://paperpile.com/c/aE78Ly/CArH). Second, small, noisy white spots were removed using SciPy’s binary_opening function (iterations=5). Third, segmentation masks which were completely black (i.e. only background) were converted to completely white (i.e. only foreground), to ensure the classifier would always receive an image with a lesion.

For classifier training, a run consisted of 20 epochs (s1 = 10 epochs, s2 = 10 epochs), with learning rates of 1e-3 and 1e-7 to 1e-5 respectively and a batch size of 16. Each of the four training datasets (HAM segmented, HAM unsegmented, ISIC segmented, ISIC unsegmented) contained the same number of images, with a 1:1 ratio between melanoma and nevus images achieved by oversampling of the minority class (melanoma) to 1254 images and undersampling of the majority class (nevus) to 1254 images. Training runs between the four different datasets did not differ, with hyperparameters, train set size and class distribution being fixed throughout.

All work was carried out in Python 3.7.4 using the fastai 1.0.60 library in combination with torch 1.4.0 and torchvision 0.5.0 [[5]](https://paperpile.com/c/aE78Ly/k4c1). Segmentation model and classifier training were carried out on a single NVIDIA GeForce RTX 2080 Ti.

**Table S2. Overview of the sensitivity and specificity for each type of classifier across the holdout, external and overall test set.** Italicized numbers indicate the higher metric when comparing between classifiers trained on a segmented/unsegmented version of the same dataset.

| **Test set components, metric** | | **Trained classifiers** | | | |
| --- | --- | --- | --- | --- | --- |
|  |  | **HAM segmented (%)** | **HAM unsegmented (%)** | **ISIC segmented (%)** | **ISIC unsegmented (%)** |
| **Holdout** | | | | | |
|  | **Sensitivity (SD)** | *90.4 (1.5)* | 87.0 (0.9) | *76.2 (2.6)* | 72.2 (5.7) |
|  | **Specificity (SD)** | 84.8 (2.1) | *91.8 (2)* | 78 (5.3) | *87.8 (1)* |
| **External** | | | | | |
|  | **Sensitivity (SD)** | *66.1 (3.1)* | 64.5 (4.7) | *84.7 (2.1)* | 70.6 (2.9) |
|  | **Specificity (SD)** | *73.7 (3.1)* | 50.7 (8.9) | 71.6 (5.3) | *84.6 (1.8)* |
| **Overall** | | | | | |
|  | **Sensitivity (SD)** | *74.9 (2)* | 72.6 (3) | *81.7 (1.9)* | 71.2 (3.6) |
|  | **Specificity (SD)** | *76.4 (2.7)* | 60.7 (6.9) | 73.2 (4.7) | *85.4 (1.3)* |

**Table S3. Overview of the sensitivity and specificity for each type of classifier across the external test set’s three individual components.** If classifiers were trained on HAM images, the first external component consists of ISIC and vice versa. Italicized numbers indicate the higher metric when comparing between classifiers trained on a segmented/unsegmented version of the same dataset.

| **External test set components, metric** | | **Trained classifiers** | | | |
| --- | --- | --- | --- | --- | --- |
|  |  | **HAM segmented (%)** | **HAM unsegmented (%)** | **ISIC segmented (%)** | **ISIC unsegmented (%)** |
| **HAM/ISIC** | | | | | |
|  | **Sensitivity (SD)** | *49.6 (2)* | 48 (7.7) | *78.2 (2.3)* | 65 (3.2) |
|  | **Specificity (SD)** | *72.4 (2.3)* | 69.8 (5.9) | 70 (9.2) | *88 (2.1)* |
| **PH2** | | | | | |
|  | **Sensitivity (SD)** | 88.5 (5.1) | *95.5 (1.9)* | *90.5 (3.7)* | 82 (2.9) |
|  | **Specificity (SD)** | *80.2 (3.8)* | 30.9 (13.6) | 82.4 (5) | *85.4 (2.8)* |
| **PROP** | | | | | |
|  | **Sensitivity (SD)** | *86.5 (6.2)* | 75.7 (1.7) | *96.2 (2.8)* | 73.5 (7.1) |
|  | **Specificity (SD)** | 55.7 (5.1) | *75.7 (8.8)* | 41.2 (5.1) | *75.7 (4.6)* |

**References**

1. [Deng J, Dong W, Socher R, Li L, Kai Li, Li Fei-Fei. ImageNet: A large-scale hierarchical image database. 2009 IEEE Conference on Computer Vision and Pattern Recognition 2009. p. 248–255.](http://paperpile.com/b/aE78Ly/kOEX)

2. [Smith LN. A disciplined approach to neural network hyper-parameters: Part 1-learning rate, batch size, momentum, and weight decay. CoRR, vol. abs/1803.09820. 1803.09820. 1803.](http://paperpile.com/b/aE78Ly/tbpC)

3. [Howard J, Gugger S. Fastai: A Layered API for Deep Learning. Information Multidisciplinary Digital Publishing Institute; 2020 Feb 16;11(2):108.](http://paperpile.com/b/aE78Ly/4ALs)

4. [Virtanen P, Gommers R, Oliphant TE, Haberland M, Reddy T, Cournapeau D, Burovski E, Peterson P, Weckesser W, Bright J, van der Walt SJ, Brett M, Wilson J, Millman KJ, Mayorov N, Nelson ARJ, Jones E, Kern R, Larson E, Carey CJ, Polat İ, Feng Y, Moore EW, VanderPlas J, Laxalde D, Perktold J, Cimrman R, Henriksen I, Quintero EA, Harris CR, Archibald AM, Ribeiro AH, Pedregosa F, van Mulbregt P, Vijaykumar A, Bardelli AP, Rothberg A, Hilboll A, Kloeckner A, Scopatz A, Lee A, Rokem A, Woods CN, Fulton C, Masson C, Häggström C, Fitzgerald C, Nicholson DA, Hagen DR, Pasechnik DV, Olivetti E, Martin E, Wieser E, Silva F, Lenders F, Wilhelm F, Young G, Price GA, Ingold G-L, Allen GE, Lee GR, Audren H, Probst I, Dietrich JP, Silterra J, Webber JT, Slavič J, Nothman J, Buchner J, Kulick J, Schönberger JL, de Miranda Cardoso JV, Reimer J, Harrington J, Rodríguez JLC, Nunez-Iglesias J, Kuczynski J, Tritz K, Thoma M, Newville M, Kümmerer M, Bolingbroke M, Tartre M, Pak M, Smith NJ, Nowaczyk N, Shebanov N, Pavlyk O, Brodtkorb PA, Lee P, McGibbon RT, Feldbauer R, Lewis S, Tygier S, Sievert S, Vigna S, Peterson S, More S, Pudlik T, Oshima T, Pingel TJ, Robitaille TP, Spura T, Jones TR, Cera T, Leslie T, Zito T, Krauss T, Upadhyay U, Halchenko YO, Vázquez-Baeza Y, SciPy 1.0 Contributors. SciPy 1.0: fundamental algorithms for scientific computing in Python. Nat Methods 2020 Mar 1;17(3):261–272.](http://paperpile.com/b/aE78Ly/CArH)

5. [Paszke A, Gross S, Massa F, Lerer A. Pytorch: An imperative style, high-performance deep learning library. Adv Neural Inf Process Syst [Internet] papers.nips.cc; 2019; Available from:](http://paperpile.com/b/aE78Ly/k4c1) <http://papers.nips.cc/paper/9015-pytorch-an-imperative-stylehigh->
